# Gender equity in Critical Care Medicine. How much have we progressed?

**DOI:** 10.62675/2965-2774.20250404

**Published:** 2025-04-02

**Authors:** Vanessa Soares Lanziotti, Kathryn Puxty, Sangeeta Mehta

**Affiliations:** 1 Universidade Federal do Rio de Janeiro Instituto de Puericultura e Pediatria "Martagão Gesteira" Pediatric Intensive Care Unit Rio de Janeiro RJ Brazil Pediatric Intensive Care Unit, Instituto de Puericultura e Pediatria "Martagão Gesteira", Universidade Federal do Rio de Janeiro - Rio de Janeiro (RJ), Brazil.; 2 University of Glasgow Department of Intensive Care Glasgow Royal Infirmary Glasgow United Kingdom Department of Intensive Care, Glasgow Royal Infirmary, University of Glasgow - Glasgow, United Kingdom.; 3 University of Toronto Department of Medicine Toronto Canada Department of Medicine, Sinai Health System, University of Toronto - Toronto, Canada.

## INTRODUCTION

The medical field has historically been male-dominated, particularly in specialties like surgery, anesthesia, and critical care medicine. Although women now enroll in medical school at rates equal to or even higher than men, gender equity in the medical profession remains elusive. Women are still significantly underrepresented in leadership positions, especially in academic medicine and specific specialties,^(1,2)^ as conference speakers, authors, peer reviewers, and guideline panel members.^(3-5)^ The gender gap in critical care medicine practice remains significant and is even more prominent in academia despite the efforts made in recent years to reduce it.^(6)^ However, to what degree have these efforts truly advanced gender equity within the critical care medicine workforce? What real advancement have we made in this field in recent years?

This viewpoint article aims to provide a comprehensive overview of the recent progress in improving gender equity within critical care medicine and to critically examine areas in which gender disparities remain prevalent, including pay gaps, leadership roles, research leadership, and funding.

## LEADERSHIP AND REPRESENTATION

Over the last few years, many studies have evaluated the impact of enhancing diversity in the workplace. They have demonstrated the benefits of diversity within medical and academic communities, as it contributes to improved scientific productivity, drives innovation, enhances impact, and even improves patient outcomes.^(7,8)^

One of the most concrete measures of gender equity in any field is the representation of women in leadership positions. In critical care medicine, there has been a gradual and slow but notable increase in the number of women taking leadership roles within professional societies, editorial boards, and academic departments. Although this represents a step towards gender equity, leadership roles are still disproportionately held by men, considering the overall proportion of women working in critical care medicine.^(9)^ When analyzing the gender of Critical Care societies past presidents, there is perhaps an increasing trend of female presidents in recent years. Among the 53 past presidents of the Society of Critical Care Medicine (SCCM) in the United States, only 11 females (21%) can be counted; however, in the last decade, 3 of 10 (30%) were women.^(10)^ Analyzing data from the *Associação de Medicina Intensiva Brasileira* (AMIB), the numbers are discouraging - from a total of 21 presidents (including the current president), only 3 were women (14%), 2 of them in the last decade, but still the majority of the board of directors have been men.^(11)^ From 1977 until today, only 2 of 19 Canadian Critical Care Society presidents have been women; however, both of these women have held this role within the past 10 years. A similar trend is noticeable in the Scottish Intensive Care Society, whereby the first 14 presidents were male, but after 27 years, the most recent 2 presidents have been female.

In recent years, many intensive care societies in America, Europe, and Asia have spoken out and promoted gender equity, publishing policy statements and declaring their commitment to reducing gender inequities. These actions are essential to improve the gender balance in leadership positions and should always be based on meritocracy and democratic processes, but opening opportunities for women to assume these positions is crucial.^(12-15)^

**Figure 1:**
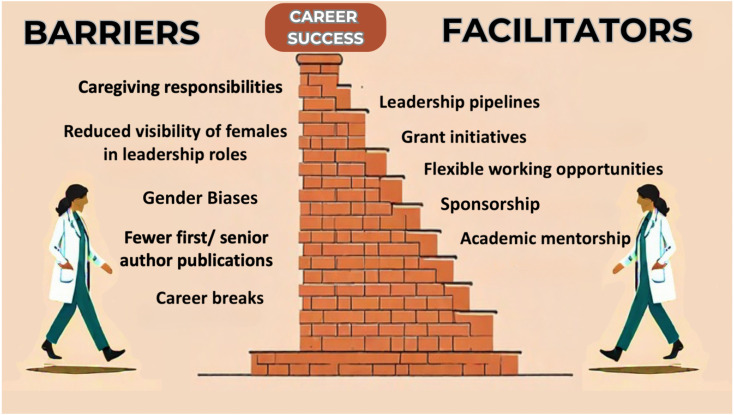
Barriers and facilitators for women to succeed in their Critical Care Medicine careers.

Other initiatives, such as introducing mentorship and leadership programs aimed at empowering women clinicians and researchers, are also important in addressing this imbalance (Figure 1). Studies have shown that female mentorship can significantly improve career progression for women in critical care. Transparent reporting of women's representation is also important to raise awareness in the intensive care medicine community and to guide and monitor goals that may be overlooked.^(9,16)^

It is important to emphasize, however, that these actions are not enough if structural obstacles such as organizational bias, lack of access to leadership pipelines, workplace culture, policy gaps, and changes on maternity rights remain intact, preventing women from advancing into leadership positions at the same pace as men.

## RESEARCH OPPORTUNITIES AND FUNDING

Obtaining grants, conducting research, and publishing in peer-reviewed journals are crucial for advancing an academic medical career. Publications significantly impact a researcher's visibility and success in securing grants, promotion, and invitations to collaborate and contribute to scholarly endeavors, including grant panels, conferences, guidelines, and editorial boards. However, gender disparities persist in critical care publications: women represent less than one-third of first authors and only about one-fourth of senior authors.^(4,5,17)^

Recent initiatives by funding bodies to promote gender equity in research grants and awards have made some progress, with an increasing number of women securing high-profile grants in critical care medicine. However, despite these advancements, the percentage of women contributing as authors in critical care publications has remained largely unchanged over the last decade.^(18)^ Some international consortia, such as the Canadian Critical Care Trials Group, better represent women authors, possibly reflecting their history of female leadership.^(19)^

## WORK-LIFE BALANCE

Work-life balance is notoriously difficult in a medical career, but for women, this balance is even more difficult, considering the multiple tasks they perform in their daily routine, including the role of mother, for example. Pregnancy and disproportionately assuming the roles of homemaker and primary caregiver for children are significant challenges, especially in a career like critical care medicine that the schedules can be more rigid and the work routine heavy. Disproportionate caregiving responsibilities and expectations placed on women exacerbate the risk of burnout, culminating in the possibility of leaving academia or giving up a career in intensive care medicine entirely.^(5,19)^

Women pursuing both medical careers and motherhood often prioritize career advancement by delaying starting a family, increasing their risks of infertility or complicated pregnancies.^(20)^

Policies aimed at improving work-life balance, including flexible working hours, parental leave, and part-time training/work options, have been introduced in some institutions. However, these policies vary, and maternity leave, for example, is far from ideal in most parts of the world. Thus, the effectiveness of these actions in reducing gender disparities and burnout rates remains unknown. Moreover, the stigma surrounding part-time employment or taking parental leave continues to disproportionately affect women, often leading to career stagnation. In academic medicine, this stagnation may be even more evident due to the reduction in scientific production in the early years of motherhood.^(20)^

## FINAL MESSAGE

Progress toward gender equity in critical care medicine over recent years is encouraging but slow, and more work is needed. Despite the progress mentioned here, gender inequality is still present. The historical and systemic gender inequities are challenging to overcome. To fully address these disparities, systemic and structural changes must be made in clinical and academic institutions, and transparency in leadership roles, research grants, and remuneration, for example. Also, cultural changes and support for women regarding maternity leave and flexible working hours, among others, are necessary. This path of change is long and requires time, investment, and willingness from the intensive care community to ensure success. Only through continuous effort and engagement with equity can we ensure that critical care medicine reflects the diversity and talent of all its professionals, regardless of gender.
